# Early Diagnosis of Cardiovascular Diseases in the Era of Artificial Intelligence: An In-Depth Review

**DOI:** 10.7759/cureus.55869

**Published:** 2024-03-09

**Authors:** Naiela E Almansouri, Mishael Awe, Selvambigay Rajavelu, Kudapa Jahnavi, Rohan Shastry, Ali Hasan, Hadi Hasan, Mohit Lakkimsetti, Reem Khalid AlAbbasi, Brian Criollo Gutiérrez, Ali Haider

**Affiliations:** 1 Internal Medicine, University of Tripoli Faculty of Medicine, Tripoli, LBY; 2 Internal Medicine, Crimea State Medical University named after S.I Georgievsky, Simferopol, UKR; 3 Internal Medicine, Sri Ramachandra Institute of Higher Education and Research, Chennai, IND; 4 Internal Medicine, Pondicherry Institute of Medical Sciences, Puducherry, IND; 5 Internal Medicine, Vydehi Institute of Medical Sciences and Research Center, Bengaluru, IND; 6 Internal Medicine, University of Illinois at Chicago, Chicago, USA; 7 Internal Medicine, University of Illinois, Chicago, USA; 8 Internal Medicine, Mamata Medical College, Khammam, IND; 9 Independent Scholar, Abi Abdullah AlDumaiti, Jeddah, SAU; 10 Health Sciences, Instituto Colombiano de Estudios Superiores de Incolda (ICESI) University, Cali, COL; 11 Allied Health Sciences, The University of Lahore, Gujrat, PAK

**Keywords:** efficacy of ai in cardiac medicine, ai in cardiology, machine learning, artificial intelligence, cardiovascular diseases

## Abstract

Cardiovascular diseases (CVDs) are significant health issues that result in high death rates globally. Early detection of cardiovascular events may lower the occurrence of acute myocardial infarction and reduce death rates in people with CVDs. Traditional data analysis is inadequate for managing multidimensional data related to the risk prediction of CVDs, heart attacks, medical image interpretations, therapeutic decision-making, and disease prognosis due to the complex pathological mechanisms and multiple factors involved. Artificial intelligence (AI) is a technology that utilizes advanced computer algorithms to extract information from large databases, and it has been integrated into the medical industry. AI methods have shown the ability to speed up the advancement of diagnosing and treating CVDs such as heart failure, atrial fibrillation, valvular heart disease, hypertrophic cardiomyopathy, congenital heart disease, and more. In clinical settings, AI has shown usefulness in diagnosing cardiovascular illness, improving the efficiency of supporting tools, stratifying and categorizing diseases, and predicting outcomes. Advanced AI algorithms have been intricately designed to analyze intricate relationships within extensive healthcare data, enabling them to tackle more intricate jobs compared to conventional approaches.

## Introduction and background

**Figure 1:**
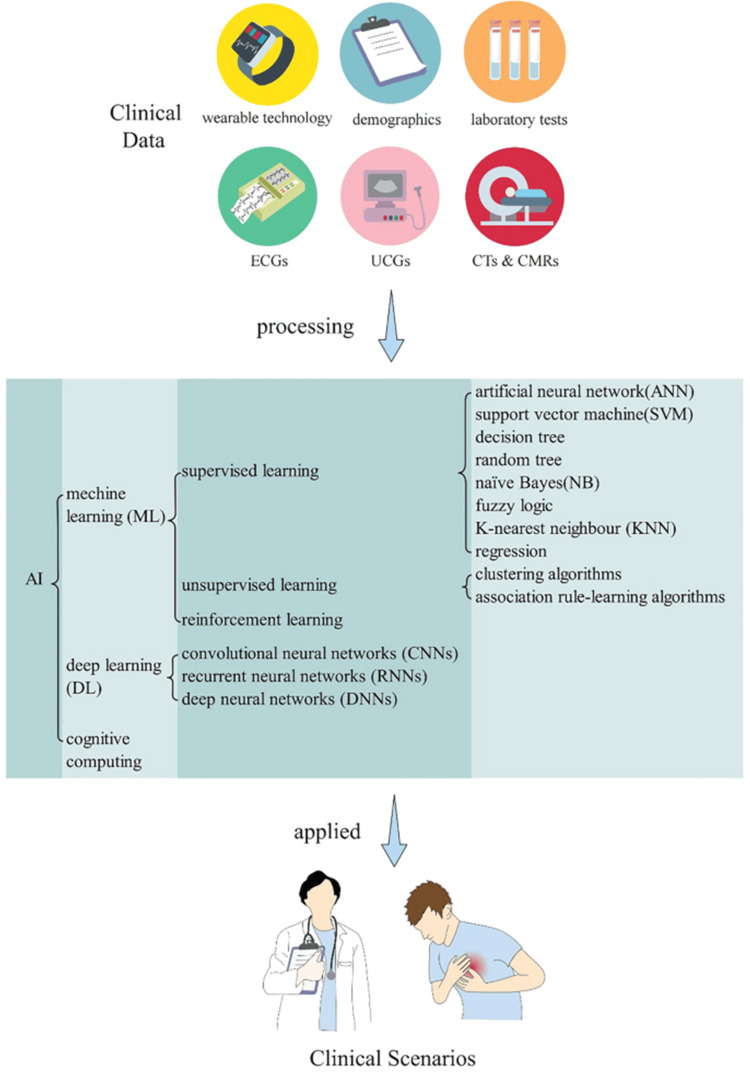
Flowchart depicting the use of artificial intelligence in clinical practice Reproduced from ref. [13] under the terms and conditions of the Creative Commons CC BY license. Copyright 2023 Springer Nature, The author(s).

Ischemia, heart failure, myocardial infarction, stroke, problems affecting the aorta and peripheral arteries, arrhythmias, and diseases of the heart valves are all examples of cardiovascular diseases (CVDs) [1]. In spite of great strides in the detection and treatment of CVDs, they remained the leading cause of death worldwide in 2022, accounting for 19.8 million fatalities [2]. Furthermore, with a daily cost of roughly $1 billion, CVDs are the most expensive sickness [3]. Despite the fact that cardiovascular illnesses are preventable, current predictions show that their prevalence will rise. By 2035, experts expect that 45% of adult Americans will have the ailment, and the yearly cost will exceed $1 trillion [4]. Numerous safe and efficient treatments are already available to combat CVD, which ranks high among public health priorities [5-8]. Over the last several years, AI's impact on CVD has been steadily increasing. The study of how computers and machine learning (ML) systems may mimic human intelligence via the use of computational techniques is known as artificial intellect. This area aims to solve human problems. A more cohesive, trustworthy, and efficient method of providing high-quality healthcare has been encouraged by the advent of artificial intelligence (AI), which provides methods for computers to mimic human cognitive functions such as learning and reasoning [9-11]. Research into the early detection and prevention of cardiovascular disorders is now underway, building on the well-established practice of using AI in cardiovascular sciences. AI consists of complex analytical tools built into computers in an effort to imitate human intelligence. ML is an AI subfield that distinguishes itself from classical mathematical algorithms by including a “learning” component gleaned from massive datasets. There has been a lot of buzz about how CVD and AI may work together to revolutionize cardiovascular health diagnostics, prognoses, and treatments. The rapid detection and diagnosis of CVDs, together with the prediction of outcomes and evaluation of prognosis, may be greatly assisted by AI. Health records and other medical equipment are good places to start when looking for real-world data on patients' conditions and the healthcare system as a whole. As a result, massive databases including quantitative, qualitative, and transactional data have been created. To analyze these datasets, AI algorithms are required [12]. AI methods that analyze massive amounts of therapeutically relevant data may help physicians make more informed clinical decisions. Also, AI may help find subclinical organ problems before they become serious. As a result, healthcare delivery is both improved in quality and efficiency [13]. Figure 1 shows a flow diagram showing how AI is being used in a healthcare setting. This study aims to summarize the current state of AI applications for cardiovascular illnesses, explore the expanding domain of AI's usage, and talk about the potential for early diagnosis and rapid decision-making.

## Review

AI in acute coronary syndrome

The acronym ACS stands for “acute coronary syndrome,” a group of medical diseases that together restrict blood flow to the heart. It encompasses a wide range of conditions, including unstable angina, non-ST-elevation myocardial infarctions, and myocardial infarctions with ST-elevation [14-16]. ACS symptoms can include severe chest discomfort that travels down the left arm. It is critical to identify ACS quickly and accurately to improve patient outcomes and reduce mortality and morbidity. The American Heart Association states that current approaches for ACS detection include discriminating between STEMI, NSTEMI, and unstable angina using electrocardiogram (ECG) and serum troponin. Cardiac troponin I (cTnI) and T (cTnT) are reliable indicators for evaluating myocardial injury, particularly in cases of myocardial ischemia [17-20]. Within 120 minutes after the discovery of ST-elevation myocardial infarction (STEMI) on an ECG, it is essential to provide perfusion and carry out primary percutaneous coronary intervention. The outcome is a 2% reduction in the mortality rate, from 9% to 7% [21]. Immediate treatment is essential since a 7.5% increase in the probability of mortality within a year occurs every 30 minutes if treatment is delayed [22].

It is crucial to remember that not all individuals with elevated cardiac markers and ST segment may really have a genuine ST-segment elevation myocardial infarction. The term “pseudo-STEMI,” which refers to STE that is not ischemic at baseline, may apply to certain people [23]. It is recommended to record many ECGs or continually monitor the ST-segment in patients without a first ST-segment elevation to avoid healthcare resources from being overloaded. Troponin production, an indicator of cardiac muscle injury, may occur in a variety of illnesses, not simply ACS. Some additional potential reasons might include heart failure or atrial fibrillation. It is also worth noting that physiological stresses or vigorous physical exercise may cause high cTn levels in otherwise healthy persons [24-27]. These restrictions have made it more challenging to correctly identify and categorize patients with suspected ACS. The consequences of underdiagnosing ACS, the limits of manual evaluation, and the complexity of diagnosis all point to the need for new approaches to diagnosis. There is hope that these techniques may improve ACS detection rates, lead to earlier diagnoses, and shorten treatment times [28].

ML has the potential to greatly aid in the timely detection of ACS [29,30]. Noninvasive evaluation of coronary artery stenosis may be possible with the application of ML methods that use computed tomography to calculate fractional flow reserve values. Research by Eberhard et al. [31] including 56 individuals with chest discomfort found that this approach was effective in 68% of cases. Acute plaque rupture was seen in 29% of patients with ACS with symptoms indicating vulnerable plaques. Revascularization was done on certain patients based on the findings of this machine-learning diagnostic tool. A practical and successful method to increase patient triage for patients with chest discomfort might be FFRCT based on ML, according to Hong et al. [32,33]. Another clinical experiment is looking at the possibility of detecting myocardial infarction in humans using a ML algorithm called myocardial-ischemic-injury-index. In this method, variables including age, sex, and levels of cardiac troponin are taken into account. Hong et al.'s study shown that this AI system can reliably classify patients as low or high risk, which enables early treatment choices that might be beneficial for them [34,35]. Myocardial ischemia patients were successfully detected by an artificial neural network (ANN) trained using the jackknife variance approach, as shown in an additional investigation. The ANN shown potential as a valuable tool for detecting ACS in patients presenting to the emergency room with chest discomfort, with a sensitivity of 88.1% and specificity of 86.2%. On top of that, different research indicated that ANN could identify NSTEMIs with a sensitivity of 90.91%, specificity of 93.33%, positive predictive value of 74.92%, and negative predictive value of 96.77%. ACS risk factors might be better served by using ANNs for the detection, monitoring, and prediction of chest discomfort [36].

**Figure 2:**
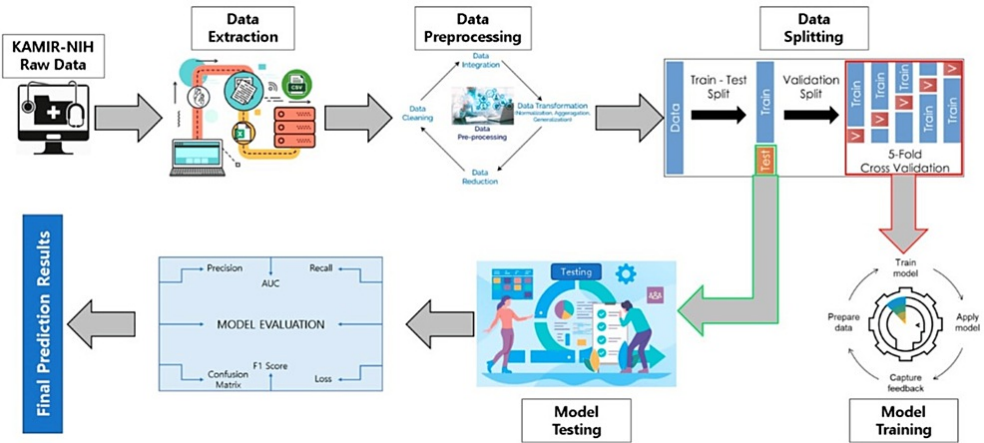
Proposed predictive modeling system's whole process Reproduced from ref. [38] under the terms and conditions of the Creative Commons Attribution (CC BY) license. Copyright 2023 by the authors. Licensee MDPI, Basel, Switzerland

Currently, ML is an innovative and advanced technique extensively used in the medical field and health informatics for diagnosing and predicting cardiovascular disorders, in particular [37]. Researcher suggested using a ML soft-voting ensemble classifier (SVEC) to predict outcomes related to ACS such as STEMI and NSTEMI, discharge reasons for hospitalized patients, and categories of mortality occurring during the hospital stay. Researcher used the Korea Acute Myocardial Infarction Registry (KAMIR-NIH) dataset, which comprises data from 13,104 individuals and includes 551 characteristics. Following data extraction and preprocessing, researcher used 125 relevant characteristics and implemented the SMOTETomek hybrid sampling strategy to address the data imbalance in minority classes by oversampling. Researcher used three ML techniques, namely random forest, additional tree, and gradient-boosting machine, in our SVEC model to predict target variables. The SVEC demonstrated superior performance compared to other ML-based prediction models in terms of accuracy (99.0733%), precision (99.0742%), recall (99.0734%), F1-score (99.9719%), and the area under the ROC curve (AUC) (99.9702%). The SVEC outperformed other models but had a slightly lower AUC than the additional tree classifier in predicting ACS outcomes. The suggested predictive model demonstrated superior performance compared to previous ML models. It is suitable for practical use in hospitals for diagnosing and predicting cardiac disorders. This allows for prompt identification of appropriate therapies and more accurate prediction of illness occurrence (Figure 2) [38,39].

 AI in cardiac arrhythmias

Bradyarrhythmia is characterized by a heart rate below 60 beats per minute, whereas tachyarrhythmia is characterized by a heart rate over 100 beats per minute [40,41]. This arrhythmia may strike anybody at any time; it shows no age bias. Normal sinus rhythm describes the heart's typical regular and predictable beating pattern. A sinusoidal origin, a controlled delay via the atrial (AV) node, a journey down the His bundle, a bifurcation into the left and right bundle branches, and finally distribution along the Purkinje fibers make up an electrical impulse. Arrhythmia begins the moment this conduction channel deviates from its normal state [42]. Atrial fibrillation is the most common arrhythmia, and it is estimated that 1.5-5% of the population may have it [43]. Because cardiac arrhythmias are often asymptomatic, diagnosing them may be challenging.

**Figure 3:**
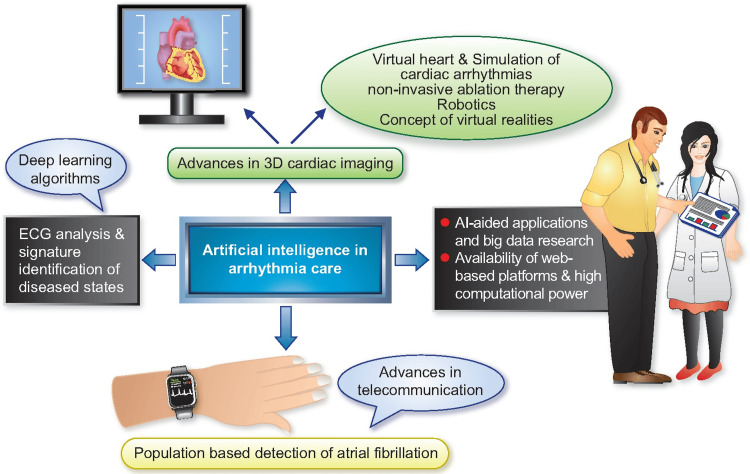
Arrhythmia treatment improved via artificial intelligence Reproduced with permission from ref. [45]. Copyright 2021 Oxford University Press, The author(s).

ECG testing may not detect atrial fibrillation in patients because the rhythm seems normal [44]. Since ECGs are now digital, AI methods for analyzing them have become commonplace, especially in the prediction of cardiac arrhythmias. In order to classify ECGs and predict when paroxysmal atrial fibrillation would commence, a very effective ML method has been developed. A sensitivity of 100% and a specificity of 95.5 % are achieved by the technique [45]. When trying to predict the likelihood of future atrial fibrillation, AI-ECG could be helpful. By analyzing the risk factors, AI may predict the occurrence of future AF episodes [46]. Timed rhythms, low-quality ECGs, tremors, alien rhythms, and noise all pose problems, as may weak or irregular P waves, which can lead to false positives when diagnosing atrial fibrillation. Recent developments in ECG feature analysis methods, noise reduction strategies, and ML algorithms have substantially improved computerized ECG interpretation (Figure 3) [47,48].

With a sensitivity of 100% and specificity of 97%, the AliveCor Heart Monitor was able to identify AF and atrial rhythm irregularities more effectively than the conventional trans telephonic monitor. An end-to-end DL methodology for ECG analysis was developed by Hannun et al. [49] and used to the analysis of ECGs to identify rhythm anomalies. Deep learning approaches, specifically end-to-end DL employing deep neural networks (DNNs), were used in this investigation. A DNN was used to detect 12 irregularities in the heart rate. Their system outperformed the typical cardiologist in identifying these rhythm abnormalities when evaluated using different data analyzed by a panel of professional cardiologists. By analyzing long-term ECG data, deep learning algorithms have successfully detected a number of cardiac arrhythmias. Identifying people at risk is made easier with AI-powered monitoring devices since they are dependable, inexpensive, and provide continuous ambulatory monitoring. This improves the detection of arrhythmias and allows for the earlier diagnosis of atrial fibrillation in patients without symptoms. One non-invasive and cost-effective way to monitor atrial fibrillation risks and control it over time is via wearable devices like smartwatches [50].

Elbey et al. conducted a study that assessed a few of these devices [51]. Wristwatch technology using single-lead ECG and photoplethysmography is just as successful as conventional methods of AF monitoring, according to nine observational studies including 1559 patients (average age 63.5 years), with 39.5% having a history of AF. On average, 75.6 days of monitoring were conducted. For the detection of atrial fibrillation, smartwatches were just as successful as composite 12-lead ECG/Holter monitoring, patch monitoring, and composite ECG monitoring systems. The sensitivity and specificity of the AF detection using smartwatches are 95% and 94%, respectively. Atrial fibrillation may be adequately monitored using either a single-lead ECG or photoplethysmography via a wristwatch. A study was conducted by Chen et al. [52] on the detection of atrial fibrillation utilizing a smart wristband that included ECG and photoplethysmography sensors.

For the purpose of diagnosing AF in patients exhibiting symptoms, the ECG-based AliveCor Heart Monitor has been the subject of substantial study. With a diagnostic accuracy of over 90% for atrial fibrillation and flutter, the AliveCor Heart Monitor has consistently shown excellent performance. Researchers created a novel technology called Cardio-HARTTM that employs bio-signals improved by AI to anticipate outcomes that are similar to echocardiography. It is possible to identify hemodynamic, functional, and structural problems using this technique. Dysfunctions of the systole and diastole are the primary foci of this functional evaluation. Additionally, this helps detect cardiac failure at an early stage. This cutting-edge technique shows improved sensitivity in detecting common heart diseases by combining ECG, polycardiogram, and a novel physiological bio-signal. The incorporation of AI enhances the efficacy of every bio-signal by detecting electro-physiological issues like arrhythmias and bundle-branch obstructions [53].

AI in heart failure

When the ventricles are unable to adequately fill or empty blood, a complex clinical disorder known as heart failure develops. This condition is accompanied by a variety of symptoms and signs. There are four classes of heart failure according to the New York Heart Association: class I, which has no restrictions on physical activity, class II, which has some restrictions, class III, which has significant limits, and class IV, which has symptoms even while at rest [54]. Improved treatments for heart failure and longer survival rates among those who suffer from the condition have led epidemiologists to declare heart failure a global epidemic. Heart failure's monetary effect on healthcare expenditures may be substantially mitigated if hospitalization rates were to be reduced. In order to reduce hospitalization expenses and improve patient outcomes, AI algorithms are used to predict which heart failure patients will experience a worsening of their symptoms [55-57]. This information is then utilized to initiate therapy at an earlier stage. Several heart failure patients were monitored with remote invasive pulmonary artery pressure during the COVID-19 pandemic. This allowed for the early detection of cardiac decompensation, which improved clinical outcomes and reduced hospital visits for these patients. The study by Stehlik et al. included monitoring the vitals of 100 heart failure patients in real time using wearable sensors that recorded their core body temperature, skin impedance, and ECG waveform [58]. Hospitalizations due to HF exacerbation were predicted with 76% sensitivity and 85% specificity using data evaluated using similarity-based modeling [59]. The results further demonstrated the potential of AI in the early detection and treatment of heart failure.

One such idea is to have patients work together with the PASSION-HF consortium's virtual doctor, Abby, to transition from professional therapy to AI-enabled personalized self-care. A self-learning feedback system, interactive physician avatar interface, decision support engine, and serious gaming tools will all be part of Abby's revolutionary features [60]. This would make self-care for heart failure more feasible, which might reduce the need for medical attention, especially in underserved rural regions where the number of doctors is expected to be lower. It is particularly important for patients to have access to tools that may help them make healthy choices outside of the hospital setting, as many health determinants are typically situated outside of it [61-63]. Help for patients suffering from heart failure is now available via the use of mHealth technologies. mHealth technologies are incredibly effective because of features like bidirectional communication made possible by interaction, customization made possible by personalization, intervention delivery made possible by timeliness, adaptation made possible by context sensitivity to individual needs, and the fact that mHealth is ubiquitous and accessible to all users. By electronically recording weight measurements and providing scheduled reminders, mHealth technologies assist heart failure patients in complying with daily weight monitoring. When patients with heart failure reach certain weight thresholds, their doctors may be informed [64].

**Figure 4:**
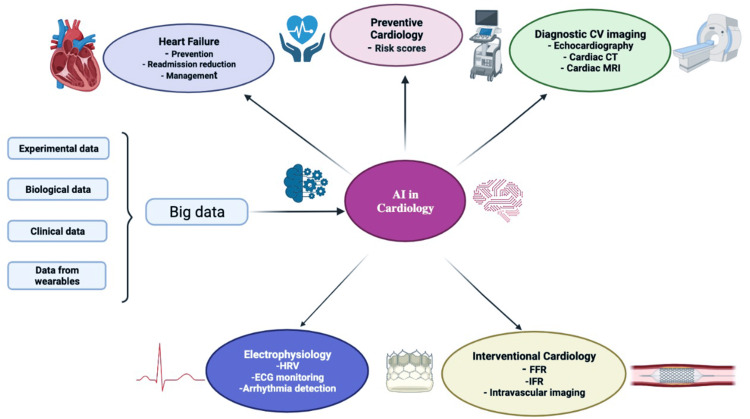
Diagram illustrating the many uses of Artificial Intelligence in the field of Cardiology Reproduced with permission from ref. [73]. Copyright 2022  Elsevier, a division of RELX India, Pvt. Ltd on behalf of Cardiological Society of India.

Not only is HF becoming more common, but it also puts a heavy financial burden on patients. Clinicians may often find it especially challenging to diagnose HF. Patients with heart failure may be classified according to their ejection fraction, which can be maintained, mid-range, or lowered [65-69]. Different approaches are necessary for treating each of these categories. As a result of fresh research and novel drug discoveries, the guidelines for the treatment of heart failure are changing at a rapid pace. AI has shown to be an invaluable resource for medical professionals when it comes to making diagnosis [70,71]. When it comes to diagnosing and treating patients, professionals rely on the Clinical Decision Support System. At a Korean tertiary hospital, researchers tested the reliability of AI-CDSS HF diagnoses in comparison to those of HF specialists using a sample of 600 patients with and without HF. Physical examination results, abnormal ECGs, left ventricular mass index, left atrial volume index, and left ventricular ejection fraction were some of the factors used by the AI-CDSS model. Based on the data, HFpEF had a 78.9% accuracy rate and no-HF had an 80.5% accuracy rate, whereas HFmrEF and HFrEF had a 100% accuracy rate. It is possible to put faith in AI-CDSS to diagnose certain forms of HF. When compared to HFrEF, HFpEF is known to have a number of underlying problems. Phenotypic mapping using ML algorithms was used in a study including 397 ambulatory HFpEF patients. Different groups of HFpEF patients were identified based on clinical characteristics, ECG values, echocardiographic findings, and outcomes (Figure 4) [72].

AI in Hypertension disease

One of the most important global public health concerns is arterial hypertension (AH). Hypertension, defined as blood pressure more than 140/90 mm Hg, is quite common and almost certain to affect 90% of the population at some time throughout their life. A substantial reduction in life expectancy has been associated with even modest increases in arterial blood pressure [74]. As a result of AH, which worsens with age, the brain, heart, and kidneys are put at serious risk from high blood pressure. Reducing worldwide rates of cardiovascular morbidity and mortality is the main objective of its treatment [75-79]. More than 580 million people, according to the study, have no idea that their blood pressure is abnormal [80-83]. Twenty years ago, clinical guidelines for AH management were vague and did not provide any recommendations for screening or treatment; now, however, they do include specific recommendations for particular high-risk categories [84-88]. ML and AI are powerful medical technologies that may aid in the accurate diagnosis of AH, the prediction of when hypertension will start, and the estimation of the possible decrease in cardiovascular events. According to recent studies, ML and AI are helping in hypertension detection, with AI reaching diagnostic accuracy rates of 80% to 90% depending on the data and model utilized. Accurate diagnosis of hypertension, which affects 9%-30% of the population, is challenging and often relies on clinical blood pressure measurements. Evidence from studies [89-94] highlights the risk and highlights the need for alternative diagnostic methods. An artificial neural network model that incorporates demographic and lifestyle variables showed great specificity but low sensitivity [59,95,96], while a classification tree ML model had limited sensitivity and specificity. Outperforming previous models, a trained k-nearest neighbors algorithm applied to ECG data attained an impressive 97.7% accuracy, 98.9% sensitivity, and 89.1% specificity. When it came to predicting hypertension status, the ECG-derived model outperformed estimates based on anthropometric, demographic, and lifestyle factors. In order to provide a more precise diagnosis of hypertension, identification technologies could aid doctors in identifying patients [97,98]. Area under the curve values in validation groups were beneficial for predictive algorithms that use data mining and ML to anticipate the risk of hypertension, such as XGBoost and Bayesian networks [99,100]. Predicting cardiovascular events using AI involves evaluating clinical parameters and visit-to-visit blood pressure fluctuations, with varying AUC performances.

Inadequate blood pressure control and lack of awareness usually exacerbate hypertension, but new methods of monitoring blood pressure could help lessen its effects. New methods that may be combined with smartphones and wearable gadgets are improving upon conventional cuff-based assessments, which are generally believed to be the most trustworthy. Among these techniques are the use of transdermal optical imaging technologies, analysis of vital signs, voice recordings, photoplethysmogram, and ECG data, and the estimation of blood pressure [101].

Some of these methods have achieved very high levels of accuracy and conformity with predetermined standards, such as those set by the British Hypertension Society and the Association for the Advancement of Medical Instrumentation, according to research [102,103]. Using voice recordings, PPG-driven models, and approaches that use ECG signals and neural networks to estimate blood pressure levels has shown promising results, meeting or exceeding clinical standards [102]. In addition, integrating auscultatory blood pressure kits with cellphones and evaluating changes in face blood flow captured by cameras on smartphones offers new ways to monitor blood pressure without intrusive procedures. The clinical accuracy of cuff-less devices remains uncertain because to the lack of validation according to International Validation Standards [104,105], despite advancements in the field. On the other hand, if AI can enhance blood pressure monitoring, it may increase patient care by relieving physicians of some of their responsibilities; this would allow them to focus more on hypertension treatment's cornerstones: patient motivation and adherence. There are benefits and drawbacks of adopting AI for hypertension detection and treatment. New research indicates that AI may improve blood pressure control and even aid in the prediction of hypertension risk factors. The field is still in its infancy, however, and some are concerned about AI's reliability, accuracy, and consistency in this domain [106-108]. By overcoming these obstacles, AI and ML may greatly enhance patient outcomes, provide personalized treatment plans, and reduce the global burden of hypertension.

AI in peripheral artery disease

A narrowing or blocking of the arteries that carry blood from the heart to the legs is called Peripheral Arterial Disease according to the CDC. Fatty plaque builds up in the arteries and causes atherosclerosis. Though it may develop in any blood vessel, peripheral artery disease disproportionately affects the lower limbs. The painful sensation of cramping in the lower legs, thighs, or buttocks while walking, known as claudication, is a common symptom of this condition. Tissue deterioration and eventual amputation are possible outcomes of this disease in its later stages. Over the years, the prevalence of peripheral artery disease has skyrocketed over the world. According to projections, 236 million people worldwide would be living with peripheral artery disease in 2015 [106]. The prevalence of peripheral arterial disease has increased more rapidly in developing nations than in industrialized ones. It is possible that the current official data on PAD may not adequately identify all forms of PAD in some regions. Inconsistent screening recommendations, a lack of understanding on both the part of patients and healthcare professionals, and the high prevalence of silent or odd symptoms in patients make it difficult for physicians to diagnose PAD. A major concern, untreated PAD may cause amputation and death. Ensuring that patients receive the proven preventative drugs and therapies they need to be healthy depends on our ability to recognize and diagnose different forms of PAD. This is an area that shows potential for AI and ML. Peripheral artery disease is one condition that can benefit from ML for early detection. Researchers demonstrated AI's effectiveness in identifying underdiagnosed PAD in a 2016 study. Clinical trial participants showing symptoms consistent with elective coronary angiography were included in the data collection. Only 17% of individuals were found to have PAD; the remaining 68% went undiagnosed. Combining various data components such as medical history, sociodemographics, genetic information, and coronary angiography findings allowed researchers to build a ML classification model. This model's main purpose is to find previously unidentified cases of peripheral artery disease. A high area under the receiver operating curve of 0.84 indicates great diagnostic accuracy, and the full model includes over 120 baseline characteristics. Classifications using traditional linear models on the same dataset yielded an AUC of 0.60, but this outcome outperforms it. With 76% sensitivity versus 56% for the previous model, the ML model showed a 19% increase in sensitivity over the traditional linear model. People with PAD who have not yet received a diagnosis may be more easily located thanks to this heightened sensitivity [107].

**Figure 5:**
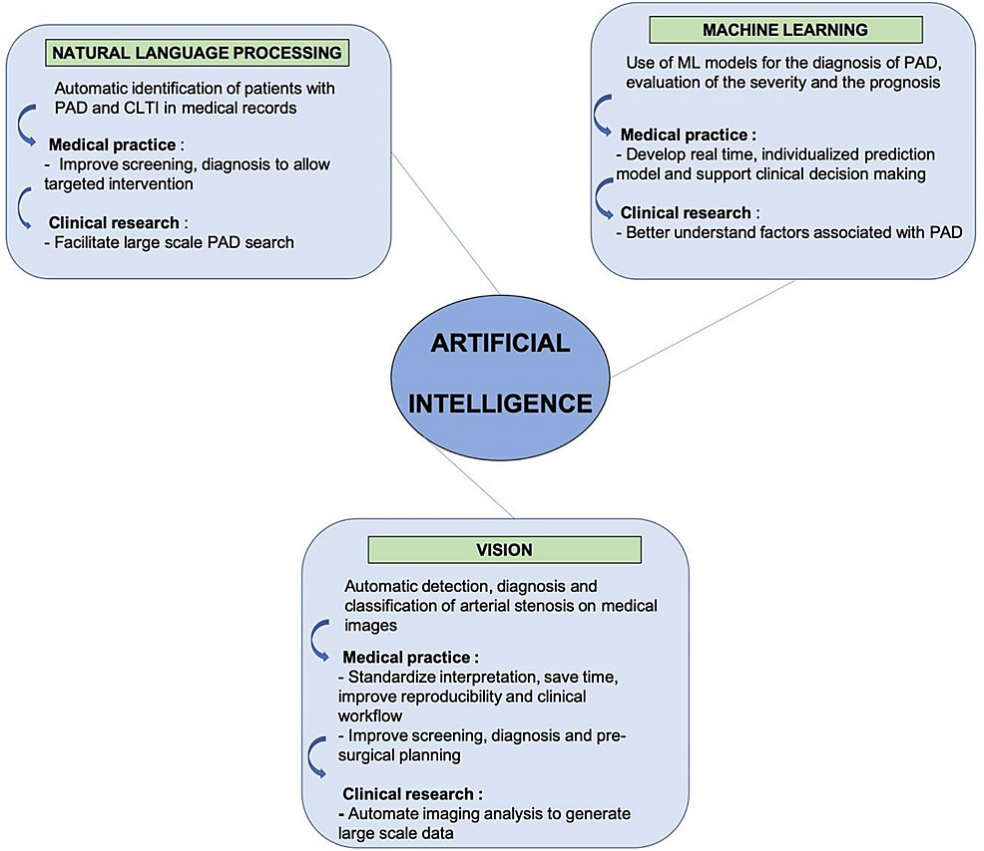
Application of AI in Peripheral artery disease Reproduced with permission from ref. [106]. Copyright 2022 by the Society for Vascular Surgery. Published by Elsevier Inc.

When it comes to PAD, ML might be used to build prediction models that help with patient prognosis evaluation and clinical decision-making. The goal of the research by Arruda-Olson et al. was to develop a prognostic tool that could provide real-time, individualised risk prediction for PAD patients by autonomously extracting data from EHRs. A larger patient pool might be available for the development of accurate risk prediction models tailored to individual patients if an automated prediction model were to gather data from EHRs. The study's findings suggest that automated, real-time risk calculators for PAD patient survival probability evaluations are feasible with the use of technological technologies. Researchers found that the developed model might save time and effort in patient care by replacing human data entry into web-based apps (Figure 5) [106-108].

AI in cardiomyopathy

Heart muscle pathology, or cardiomyopathy, is a medical disorder that causes abnormalities in the heart's structure and function, as well as abnormalities in its electrical activity. Heart failure, along with significant illness and mortality, is a common symptom of cardiomyopathies, a group of related illnesses. Out of every 100,000 adults, five will have dilated cardiomyopathy, whereas 0.57 will be affected by this condition in children. After hypertension and coronary artery disease, it is the third leading cause of heart failure in Americans. Two primary clinical manifestations of cardiomyopathy-dilated cardiomyopathy and ischemic cardiomyopathy are known to cause heart failure. In dilated cardiomyopathy, the left ventricle enlarges and contrac.tions are impaired. In ischemic cardiomyopathy, the heart's oxygen supply and demand are chronically out of sync, causing myocardial scarring, cell death, and ventricular failure. Both DCM and ICM play important roles as risk factors for the development of HF, hence it is necessary to diagnose and classify them promptly. To diagnosis cardiomyopathies, a variety of clinical methods may be used, such as ECG, cardiac magnetic resonance imaging (MRI), cardiac computed tomography scan, blood tests, and echocardiography. However, the diagnostic classification and characterization of these disorders is not consistent [109-119].

MRI and echocardiography are the gold standards for diagnosing LVH, a hallmark of hypertrophic cardiomyopathy [120]. ML, DL, and cognitive computing are sub-disciplines of AI. ML, a branch of AI, has shown promising results in several areas of medicine, including the detection of new genotype-phenotype associations, the improvement of illness diagnosis, the mitigation of adverse effects, and the decrease of hospital readmission and death rates. Differentiating between diseases and training ML models to forecast disease subtypes are two potential uses of RNA-Seq data. The first study demonstrating the efficacy of ML modeling on whole genome transcriptomic data for clinical cardiomyopathy diagnosis was carried out by Alimadadi et al. using five ML algorithms-svmRadial, pcaNNet, decision tree, ENet, and random forest-this study analyzed RNA-Seq data from 49 nonfailure controls, 47 patients with DCM, 47 patients with ICM, and 41 patients without DCM. An early ML model achieved an accuracy of roughly 93% when trained on svmRadial to differentiate between NF and DCM, 82% when trained on RF to differentiate between NF and ICM, and 80% when trained on ENet and svmRadial to differentiate between DCM and ICM [118]. To retrain ML models, 50 highly contributing genes were chosen as potential biomarkers to detect NF and DCM, 68 HCGs for NF and ICM classification, and 59 HCGs for DCM and ICM classification. When it came to differentiating between NF and DCM, the retrained models achieved an accuracy of over 90% with Random Forest, over 90% with pcaNNet, and about 85% with pcaNNet and Random Forest.

In cardiac dysfunctions such as cardiomyopathies, hypertrophies, and fibrosis, the pathway analysis implicates the chosen highly connected genes. One of the long-standing uses of AI in ECG systems is automated analysis of readings. However, not all results are precise or applicable to treatment. There are a number of new ways that have emerged as a result of AI research that may significantly change the way the ECG works. The classification of ECG data in traditional automated ECG interpretation relies on criteria that have been set by humans. Using reference ranges based on demographic data is a frequent technique when creating these standards. An ECG may measure how often PR intervals occur. The computer has determined that a PR interval greater than 200 ms indicates first-degree heart block. A complicated set of diagnostic criteria may be developed from a number of input ECGs via the deep learning approach, which may uncover relevant aspects [121,122].

The researchers at the Mayo Clinic used a convolutional neural network deep learning method that is based on AI to detect abnormalities in the left ventricle's systolic function. Second, after examining data from 44,959 individuals with 12-lead ECG and echocardiography data, they used a second sample of 52,870 patients to validate their strategy. It was successful for the network model with an AUC of 0.93, 85.7% certainty, 85.7% accuracy, and 86.3% sensitivity. By combining AI with ECG, asymptomatic persons may be identified for ALVD. Mayo Clinic-funded research by Shrivastava et al. developed a CNN model for early DC detection in ECGs. With an Area Under the Curve of 0.955 in a sample of 16,025 healthy adults and 421 patients with Dilated Cardiomyopathy, AI-ECG was able to identify LVEF≤45%. It is possible to screen for Dilated Cardiomyopathy and decide whether patients need further echocardiographic testing using the AI-ECG since it has a negative predictive value of above 99%. Those with normal left ventricular ejection fraction but diastolic dysfunction may be able to be detected by an ECG driven by AI, according to the study's authors. Ko et al. developed and assessed an AI-enabled ECG using a dataset including 24,48 patients with hypertrophic cardiomyopathy and 51,153 healthy controls who were matched for age and gender. An ECG was used to diagnose HCM in a test group of 612 patients with HCM and 12,788 healthy controls. The model achieved an area under the curve of 0.96, sensitivity of 87%, and specificity of 90%. A promising indication that AI-ECG might be useful for screening hypertrophic cardiomyopathy [123] is the model's excellent performance in subjects less than 40 years old.

Siontis et al. demonstrated that AI-ECG can accurately distinguish between a typical 12-lead ECG and juvenile HCM in a group of 300 patients with HCM by using a deep-learning AI model. There were 18,439 non-HCM controls and patients matched according to age and sex. The case group had a higher average AI-ECG probability of hypertrophic cardiomyopathy (92% vs. 5% in the control group). The AI-ECG model achieved an area under the curve of 0.98 for HCM detection, with a sensitivity of 92% and a specificity of 95%. There was a 22% positive predictive value and a 99% negative predictive value. Patients of both sexes, including those with positive and negative genes for hypertrophic cardiomyopathy, showed similar performance with the model. Performance would often enhance with time. Area Under the Curve for the test in children less than 5 was 0.93. According to the study, the AUC was 0.99 for the 15-18 age bracket [119,124].

Cardiomyopathy is most common during pregnancy and the first few weeks after giving birth. In comparison to natriuretic peptides and traditional clinical indicators, ECGs enhanced with AI performed better, according to a study conducted by the Mayo Clinic. Using ECG data, a deep learning model was trained to diagnose cardiomyopathy in 1,807 women. Seven percent of these individuals exhibited a left ventricular ejection fraction below 35%, 10% below 45%, and 13% below 50%. By analyzing the ECG, a deep learning model was trained to detect cardiomyopathy when the Left Ventricular Ejection Fraction was below 35%, 0.89 when it was below 45%, and 0.87 when it was below 50%. Black women's AUC was 0.95 and Hispanic women's was 0.98 among those with LVEF levels of 35% or lower; White women's AUC was 0.91. Natriuretic peptides ranged from 0.85 to 0.86 in the multivariable model, which had an area under the curve of 0.72 [125].

Echocardiography provides a visual representation of the heart. The accuracy of echocardiographic tests is directly correlated to the competence of the operator, who in turn needs extensive training to become proficient. These days, AI is used in echocardiography to automate tasks like measuring strain, calculating ejection fraction, and evaluating left ventricular function. Marketers have created real-time guidance software to assist less experienced physicians in obtaining normal echocardiographic images. A convolutional neural network achieved a 92% success rate in identifying eight standard 3D echocardiographic images, when tested against ground truth training data annotated by doctors. A non-cardiologist can conduct an echocardiogram in an emergency; this might aid in the education of cardiac sonographers and the mechanization of image acquisition. Using a deep learning model, Zhang and colleagues were able to achieve C statistics of 0.93 for hypertrophic cardiomyopathy, 0.87 for cardiac amyloidosis, and 0.85 for pulmonary hypertension. In addition, they proved that it could differentiate between different echocardiographic perspectives and reliably automate cardiac structural evaluations that were comparable to or better than human measurements [126,127].

Hwang and colleagues developed a deep learning algorithm using convolutional neural networks for long short-term memory to differentiate between hypertrophic cardiomyopathy, cardiac amyloidosis, and hypertensive heart disease, which are common causes of left ventricular hypertrophy. The area under the curve for hypertensive heart disease (0.962), heart failure (0.982), and cardiac amyloidosis (0.996) were all found in the test set. Compared to echocardiography experts, whose accuracy rates were 80.0% and 80.6%, the DL algorithm achieved a much higher 92.3% [127,128].

Since the turn of the century, cardiac MRI has come a long way in terms of patient diagnosis and prognosis. The left and right ventricles of the heart can be reliably evaluated using CMR, which also helps in accurately diagnosing the condition's underlying cause by identifying tissue characteristics using late gadolinium enhancement, T1, and T2 mapping. Cardiomyopathies can be diagnosed and prognostically assessed with the use of cardiac MRI, according to a study on the subject [128].

A technique called late gadolinium enhancement uses the prolonged presence of gadolinium-based contrast agents to differentiate scar tissue, necrosis, or inflammation from healthy tissue. If there is myocardial edema or damage, T2-weighted imaging can show it. Over the course of three years, Bruder et al. tracked the all-cause and cardiac mortality rates of 243 patients diagnosed with hypertrophic cardiomyopathy. A total of 67% of cases had LGE, which was associated with an OR of 5.5 for all-cause mortality and an OR of 8.0 for cardiac mortality. There is a noticeable pattern of patchy distribution of late gadolinium enhancement in approximately two-thirds of patients with hypertrophic cardiomyopathy, particularly at the right ventricular septal insertion sites and in the areas with the most hypertrophy. Late gadolinium enhancement was associated with an eightfold increase in heart failure, appropriate implantable cardioverter-defibrillator activation, and cardiac mortality in a study of 65 patients with dilated nonischemic cardiomyopathy and an ejection fraction of 35% or less. Results showed that patients with dilated cardiomyopathy who showed midwall fibrosis had similar results to those with ischemic cardiomyopathy, when compared to 161 patients with ischemic cardiomyopathy. The study included 97 patients with DCM. If scar tissue or fibrosis is present, it suggests a poor prognosis and a less than ideal response to device therapy, just like in ischemic cardiomyopathy [129].

 AI in congenital heart disease

Problems with the heart's structure that are noticeable from birth are known as congenital cardiac disease. There are a variety of minor to severe symptoms that might appear, and the worldwide incidence of this disorder varies from 0.8% to 1.2%. Less severe, more common, and potentially self-correcting, minor cardiac abnormalities include pulmonary stenosis, patent ductus arteriosus, atrial and ventricular septal defects, and ventricular and atrial septal defects. Surgery is often necessary for babies with more serious defects, such as tetralogy of Fallot, within their first year of life due to the increased risk they provide. There has been a dramatic increase in the number of persons with congenital cardiac defects, with over 90% of neonates surviving into adulthood, because to advancements in operations and drugs. Unfortunately, surgery cannot cure the disease. As adults, people with even minor surgical defects may have complications. Prenatal diagnosis of congenital heart disease significantly improves results for both the newborn and their care in the long run, according to the available research. AI has the ability to assess data from many imaging modalities and complex medical diagnoses in order to develop algorithms that may enhance prenatal diagnosis, adult disease therapy, and outcome prediction [130-134].

Prenatal diagnostics using AI algorithms to automatically acquire standard cardiac imaging planes might one day be a thing of the past, according to research. Because of this, physicians are able to save time and yet get high-quality images for analysis. By feeding 2,694 participants' mid-trimester ultrasound scans into a Convolutional Neural Network-a deep learning AI that detects and labels important patterns in pictures - Baumgartner et al. demonstrated this successfully [135]. In routine screening, the algorithm produced high-quality fetal cardiac imaging planes, proving that AI may be effective with small data sets [135]. In their study on prenatal diagnosis, Yang et al. demonstrated that AI had a higher detection accuracy. We found a detection accuracy of 73.8% for atrial septal defect, 78.8% for ventricular septal defect, and 88.9% for patent ductus arteriosus out of 518,258 people who were analyzed. When compared with auscultation, the detection accuracies for ventricular septal defect (p=0.007) and patent ductus arteriosus (p=0.021) were significantly higher [136].

Prognosis and risk categorization for adult CHD patients is sometimes a lengthy and complicated process; however, AI has the potential to streamline and improve this process. Patients who had undergone repair for Tetralogy of Fallot were categorised as having a low, moderate, or high risk for ventricular arrhythmias based on a risk assessment provided by a ML algorithm using clinical data. Using cardiac MRI datasets, deep learning algorithms were used to predict life-threatening events such as tachycardia and cardiac arrest. Using information from more than 235,000 people who had congenital heart surgery, models were developed to forecast variables such as the likelihood of death and the duration of hospitalisation. These models show that AI can predict all possible risk factors for CHD, as long as the data is big and diverse enough for algorithms to handle [135-137].

Without large data sets and ongoing research to train algorithms, implementing AI in practice is very difficult. Studies on coronary heart disease often use small samples, which limit the generalizability of the results and make it difficult for algorithms to learn and interpret. If we want AI datasets to be more efficient and effective, we need to work together and upgrade our infrastructure. Also, doctors and data scientists do not talk much about patients' demands, search terms, and how AI can help with such things. Working together, computer scientists and medical professionals may better understand one another's disciplines. Doctors, nurses, and other medical staff should be familiar with AI's foundational concepts and open to using them to save time and effort. If computer scientists are interested in using AI to treat congenital heart disease, they should be well-versed in medical procedures and have ideas for AI-friendly solutions [135-138].

AI in valvular heart disease (VHD)

VHD refers to problems with the aortic, mitral, tricuspid, or pulmonary valves. Clinically significant VHD affects around 10% of the population over 65. The identification and treatment of cardiovascular illnesses might be significantly improved with the application of AI [139]. VHD is very dangerous; hence it is critical to diagnose and treat the condition as soon as possible. Various forms of echocardiography, phonocardiography, and ECGs have used AI to assist in the detection of VHD [140]. When it comes to VHD, low- and middle-income countries are more affected by rheumatic heart disease, while high-income nations are more impacted by degenerative illnesses. In any case, the global incidence of VHD has increased by 45% in the last 30 years due to the ageing population [141]. There is no requirement for specialized equipment when employing AI for ultrasound screening of aortic stenosis [142]. AI-enabled digital stethoscopes could be useful for identifying heart murmurs. Although computer-assisted auscultation may detect rheumatic heart disease early on, it is more often acquired in poor countries [143]. The progression of aortic valve disease may be assessed using AI-based algorithms that integrate data from echocardiographic valvular assessment with additional clinical information [144]. There is a wealth of data available for AI applications in biochemical test results and clinical assessment data. With the use of AI, it may be easier to acquire and segment pictures of cardiac and valve structures for research purposes [145]. Commercially available digital stethoscopes and computer-aided auscultation both improve the identification and classification of heart murmurs. Automated evaluations of several parameters, pattern detection, and photo sorting are all ways in which AI may improve image quality. Personalized treatment regimens and disease monitoring might both benefit from solutions offered by ML. Transcatheter valve replacement decisions, such as size and selection, may be improved with the use of AI by automating the evaluation of anatomical dimensions derived from imaging data [146]. The presence of mitral regurgitation may be detected by an AI system using a single lead ECG [147]. AI has been used in echocardiography for a variety of purposes, including picture capture, image classification, quality assessment, and the detection of cardiac amyloidosis. A deep learning framework may be used to screen for VHD and, if detected, to segment in order to ascertain severity. The use of ML to classify the severity of AS in echocardiographic data could help in deciding when to replace the aortic valve. Natural language processing has been used to predict severity from echocardiography reports due to the challenges in obtaining and using structured data generated by echocardiographers. There is a 99% PPV and NPV for NLP. More and more, VHD is turning to ML to forecast AVR results and reactions. Predicting death in patients with severe AS undergoing AVR using the random forest approach inside the supervised ML method is possible [148]. The multidata integration and prediction capabilities of AI are really remarkable. These capabilities include genetic, imaging, electrophysiological, and clinical data, among others.

By analyzing large amounts of data, they facilitate the rapid decision-making process. Similar to how the visual cortex in the brain is structured, convolutional neural networks are able to identify images by identifying key features inside them. By using filters on the left ventricular outflow tract and the left atrium, respectively, to extract features from pictures and correlate them with outcomes of interest, it has been feasible to localise regurgitation for atrial fibrillation and mitral regurgitation. Finding and tracking the mitral and tricuspid valve leaflets in the apical four-chamber view allows AI to identify illness. Automated size and function measurements are the goal of applying image segmentation to 2D and 3D echocardiographic chamber images [149]. Very accurate VHD screening is provided by ECGs that are driven by AI. However, its low PPV emphasizes the need for a hybrid strategy including clinical judgement, especially in primary care settings [150].

Limitations of AI

Despite its limitations, AI has achieved remarkable strides in a number of medical domains. The importance of data bias and an equitable algorithm. The quality of the training data is the primary determinant of the prediction accuracy in supervised learning. This situation necessitates the precise classification of the real data. Accurate labelling of the training data pictures is crucial for an image classifier to work well. The same category also includes data bias. It would be inappropriate to use an x-ray classification algorithm developed for use with male data on females. Data bias may be subtle and not always easy to spot. Because it is always evolving, dynamic data may cause major issues. Included in this group is algorithmic fairness. Prejudice against underprivileged populations might be a result of algorithms educated on historical data. An outstanding example is the publication of an algorithm for the management of the health of millions of patients. Black patients were falsely shown by the algorithm to be much sicker than white patients [151].

Malicious inputs used to deliberately manipulate ML algorithms in order to cause misclassification and introduce security holes is an example of an adversarial attack. The risk is real, and proper precautions are currently being prepared, even though no incidents have been documented [152]. Recognising that, despite the increasing use of AI algorithms in research, the most trustworthy approach in medicine is still the randomised clinical trial that evaluates the algorithm's influence on patients' clinical outcomes is essential when thinking about the effect on clinical outcomes. Just being in harmony with the fundamental reality is not enough. The therapeutic outcomes of RCTs ought to be at least as good as, if not better than, what would be obtained by using human intelligence [151,152].

## Conclusions

In this narrative review, we have examined a wide range of literature on the application of AI in the early detection of CVDs. Summarily, AI techniques have shown great potential in improving the accuracy, efficiency, and timing of CVD diagnosis. By combining ML algorithms with image analysis and other AI techniques, risk factors for CVDs can be detected early, thereby enabling early intervention and personalized treatment plans for patients. The net result will be an improvement in diagnostic capabilities, a reduction of diagnostic errors, and adequate management of health resources. However, it is important to note the limitations mentioned above as they call for further research. Moving forward, there should be a focus on validating the efficacy and reliability of AI-based diagnosis in diverse patient populations and healthcare settings. Ethical issues should also be addressed to ensure that AI is used appropriately and fairly in medical practice. This review emphasizes the importance of AI in the management of CVDs, and we anticipate that the knowledge acquired will help us improve healthcare delivery.
